# Drastic power factor improvement by Te doping of rare earth-free CoSb_3_-skutterudite thin films†

**DOI:** 10.1039/d0ra02699a

**Published:** 2020-06-03

**Authors:** Cédric Bourgès, Naoki Sato, Takahiro Baba, Tetsuya Baba, Isao Ohkubo, Naohito Tsujii, Takao Mori

**Affiliations:** WPI-MANA, CFSN, National Institute for Materials Science (NIMS) Namiki 1-1 Tsukuba 305-0044 Japan MORI.Takao@nims.go.jp

## Abstract

In the present study, we have focused on the elaboration of control of Te-doped CoSb_3_ thin films by RF magnetron sputtering which is an attractive technique for industrial development of thermoelectric (TE) thin films. We have successfully synthesized sputtering targets with a reliable approach in order to obtain high-quality films with controlled stoichiometry. TE properties were then probed and revealed a reliable n-type behavior characterized by poor electrical transport properties. Tellurium substitution was realized by co-sputtering deposition and allowed obtaining a significant enhancement of the power factor with promising values of PF ≈ 0.21 mW m^−1^ K^−2^ near room temperature. It is related to the Te doping effect which leads to an increase of the Seebeck coefficient and the electrical conductivity simultaneously. However, despite this large improvement, the properties remained far from the bulk material and further developments are necessary to improve the carrier mobility reduced by the thin film formatting.

## Abstract 2

In the present study, we have focused on the elaboration of control of Te-doped CoSb_3_ thin films by RF magnetron sputtering which is an attractive technique for industrial development of thermoelectric (TE) thin films.

## Graphical abstract

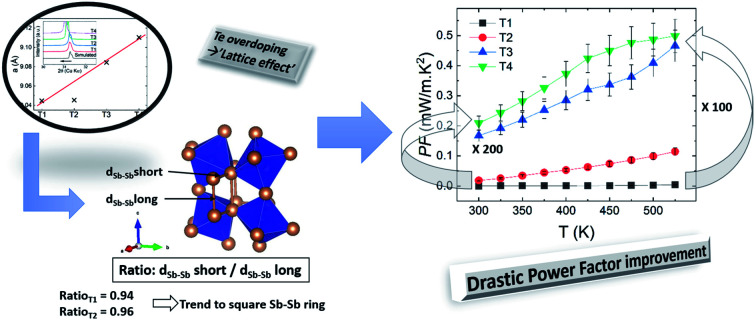

## Introduction

Thermoelectricity represents a technological gateway in the current research dynamic focused on sustainable energy development. Through appropriate devices, thermoelectric (TE) materials are attractive for dynamically harvesting energy from surroundings, like body heat, to power Internet of Things (IoT) applications.^1,2^ For such applications, the development of thermoelectric materials characterized by large TE performance near room temperature is required in addition to preferable flexible formatting. Significant power harvesting from waste heat requires TE material with a high power factor (PF) and low thermal conductivity.^3^ The TE performance of those materials is quantified by the dimensionless figure of merit *zT* = PF × *Tκ*^−1^ = *S*^2^*σTκ*^−1^ (*T*, absolute temperature; *S*, Seebeck coefficient; *σ*, electrical conductivity; *κ*, thermal conductivity). These properties typically have a tradeoff relationships, and several strategies have been investigated, such as various nanostructuring,^4,5^ utilizing interfaces and composites,^6–8^ magnetism,^9–11^ and topological states,^12^ to bypass these tradeoffs and enhance the *zT*.

For the past more than two decades, skutterudite CoSb_3_ has been considered as one of the most promising TE material mostly for mid-temperature (300–800 K).^13–16^ Its high carrier mobility and suitable Seebeck coefficient lead to attractive PF, even near room temperature, and both conduction types (p and n) are achieved. Moreover, its relatively complex cubic structure contained two icosahedra voids formed by the CoSb_3_ octahedra which allowed the introduction of a foreign element M for acting as a dopant or/and promoting intrinsic low thermal conductivity. Many efforts have been done to obtain further improvement in the electrical transport properties and reduce the lattice thermal conductivity, such as doping and nanostructuring.^14,15,17–21^ More recently, several studies have reported the thin films synthesis of CoSb_3_ skutterudite in order to exploit the effects of interface and grain boundary in the low-dimensional system, that can effectively scatter phonons, for promoting low thermal conductivity.^22–34^ In fact, TE generator based on thin films is attracting much attention for potential IoT applications as sensors, micro-power sources, flexible devices.^1,2^ Various thermoelectric materials composed of abundant elements have been fabricated into thin films. For example, oxides and some very high performance thin films has been recently reported with Heusler compounds, for example.^35–37^ Although some promising results have been published regarding CoSb_3_ skutterudite thin films, the electrical properties remain lower than those of bulk materials due to the difficulty in controlling element composition during preparation.^22,29,31,33^ Guest ion (rare-earth or alkali-earth metal)-free skutterudites are suitable for thin films growth because they are not easily oxidized.^27^ Since guest ion plays an important role in lowering thermal conductivity, we need to reduce the high thermal conductivity of guest ion-free skutterudite by other methods.^29,30^ Substitution of Sb by semimetals elements is a utilized strategy to affect the electronic structure of the CoSb_3_ skutterudite by increasing the carrier concentration/electrical conductivity, in addition, to reduce the lattice thermal conductivity by phonon-electron scattering enhancement. Up to this date, several studies on Te-doped or Si-doped CoSb_3_ materials have been made in bulk material and seem effective to enhance the electrical and thermal properties.^17,21,38–41^ Fabrication of thin films samples based on these materials can be expected to benefit us to obtain further improved rare-earth free skutterudite materials.

In the present study, we were focused on the elaboration of rare earth-free CoSb_3−*x*_Te_*x*_ skutterudite thin films by magnetron sputtering which is one of the numerous techniques for preparing thin films at an industrial scale. We have realized CoSb_3_ target for sputtering with a reliable approach for controlling the raw material parameters. Then CoSb_3−*x*_Te_*x*_ thin films were deposited by co-sputtering using CoSb_3_ and pure Te targets. Different power was used for the Te target in order to obtain different doping levels and optimize the electronic and thermal transport properties simultaneously.

## Experimental procedures

### Target preparation

For the CoSb_3_ target, pure elemental Co (powder, ≥99.9%, ≤150 μm, Aldrich) and Sb (powder, ≥99.9%, −100 mesh, Aldrich) were weighed according to the nominal composition of CoSb_3_. The elemental powders were mixed and ground by hand milling in an agate mortar and sealed in a quartz tube under a dynamic vacuum. The mixture was melted/quenched at 1423 K in an electric furnace and annealed 4 days at 973 K. The obtained powder mixture was sieved through >212 μm and loaded into a graphite die with a diameter of 50 mm coated with carbon paper. Densification by spark plasma sintering (SPS – 511S, Fuji Electronic Industrial) was processed at 773 K for 5 min (heating and cooling rate of 50 K min^−1^) under a pressure of 40 MPa and vacuum enclosure.

For the Te target, a high purity commercial Te target (99.99%, Furuuchi Chemical Company) was used.

### Film preparation

CoSb_3_ skutterudite thin films were prepared by RF co-sputtering deposition technique *via* AVC-corporation double chamber magnetron sputtering ultrahigh vacuum system. The Pyrex glass was chosen as substrates, which were cleaned by using ultrasonic cleaning for 10 min in acetone, 10 min in absolute ethyl alcohol, and 10 min in deionized water, respectively. The chamber was pumped down to 5.0 × 10^−6^ Pa and the working pressure was kept in 1.0 Pa with Ar flow of 6 cm^3^ min^−1^. All the samples were deposited with the same sputtering power of 50 W for the CoSb_3_ target and 0 W, 5 W, 7 W, and 9 W were used for the Te target, respectively, for T1, T2, T3, and T4 film. The sputtering time was set to 3 h at 573 K, then annealing steps of 1 h were taken at the same temperature for preventing the film to peel off. The resulting deposition rates increased with the sputtering power of the Te target and are estimated to be 0.087, 0.088, 0.093, and 0.104 nm s^−1^ respectively for T1 (0 W), T2 (5 W), T3 (7 W), and T4 (9 W) (S1). Second annealing was realized at 523 K for 1 day under vacuum for crystallizing the skutterudite and improving the films homogeneity. Two substrates were done for each deposition and a second series with a new CoSb_3_-target was deposited with the same parameters to confirm the results reliability on 4 samples for each condition.

### Film characterizations

The crystal structure was investigated by X-ray diffraction (XRD) technique with the prescriptive *θ*–2*θ* mode with the angle of 2*θ* = 10°–100° (Smart Lab3 Rigaku Corporation). X-ray powder diffraction patterns were refined by Rietveld analysis using the FullProf and WinPLOTR software packages.^42,43^ The shape of the diffraction peaks was modeled using a Thompson–Cox–Hastings pseudo-Voigt profile function.^44^ Zero-point shift, asymmetry parameters and lattice parameters were systematically refined, and the background contribution was manually estimated. The surface morphology was analyzed by scanning electron microscopy (SEM) (Hitachi SU-8000) and the component analysis was proceeded by energy dispersive spectroscopy (EDS). The thickness of the thin films was obtained by using a Dektak 6M Stylus Profiler measurement system. The carrier concentration and mobility were obtained from Hall effect measurements (Resitest 8300). The electrical conductivity (*σ*) and Seebeck coefficient (*S*) were simultaneously measured by the four-probe method from 300 K up to 525 K using a ZEM-3 (ULVAK Advance-Riko) device under partial helium pressure. The estimated measurement uncertainties are fixing to 6% for the Seebeck coefficient and 8% for the electrical conductivity.^45^ The cross-plane thermal conductivity was evaluated by using a picosecond time-domain thermoreflectance (TD-TR) instrument (PicoTR, Picotherm Corp.) in a front-heating/front-detection configuration. A 100 nm-thick Pt thin film was deposited on the CoSb_3_ film surface by using a DC sputtering system to detect transient temperature changes. A 1550 nm infrared pulsed laser with a repetition frequency of 20 MHz and a pulse duration of 0.5 ps was used as a heat source. A 780 nm probe laser was used to detect the thermoreflectance signal. The picosecond TD-TR system was customized to reduce the spot size of the probe laser to *ca.* 5 μm as described elsewhere.^46^ The improved mirror image method for fitting all the range of pulse interval^47,48^ was used to determine the thermal conductivity value. Here, we assumed the specific heat value of all films as 3*R* from Dulong–Petit law (0.235 J g^−1^ K^−1^).

## Results and discussion

**Fig. 1:**
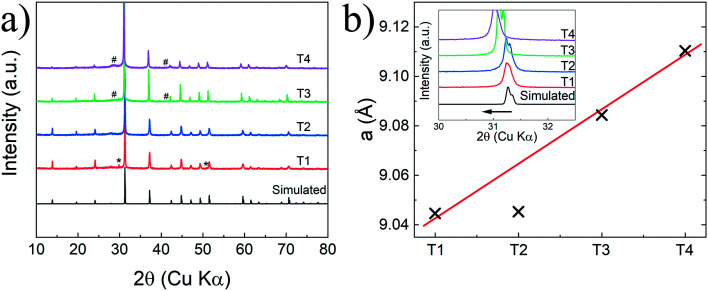
(a) XRD patterns recorded at room temperature of the CoSb_3_ doped Te thin films (T1, T2, T3, and T4) and the simulated CoSb_3_ pattern. The (*) attest to the presence of free-Sb reflexion and (#) to the low-intensity peak (015) and (110) of the Sb_2_Te_3_ nanoparticles, and (b) evolution of the lattice parameter *a* and the highlight of the (310) reflexion shift (inset) with the Te target power (the red line is just a guide to the eye).

**Fig. 2:**
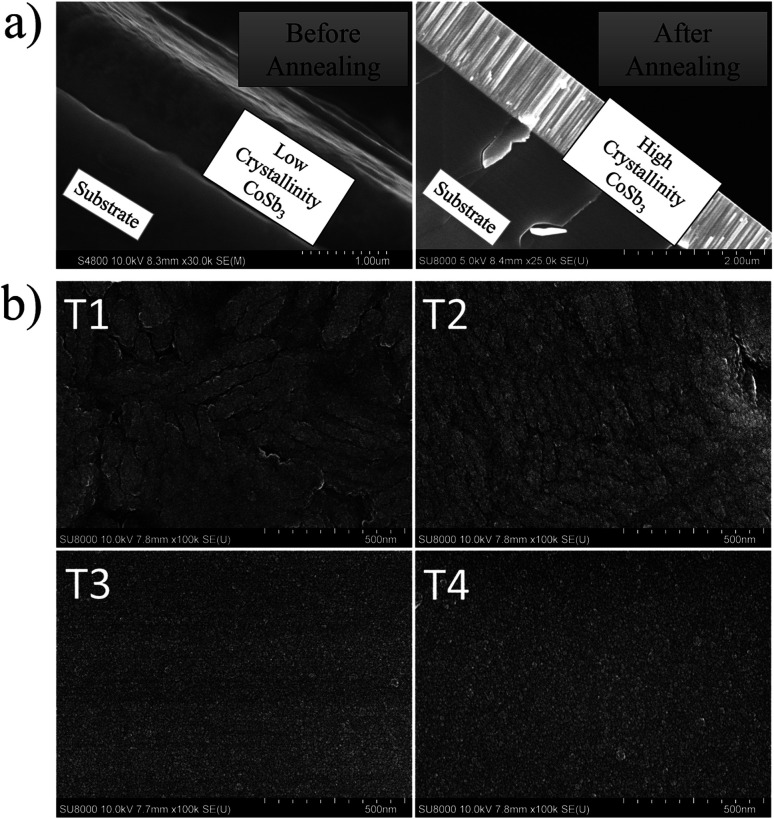
(a) Cross-section SEM image of the undoped CoSb_3_ thin film before and after annealing and (b) surface morphology of the CoSb_3_ doped Te thin films (T1, T2, T3, and T4).

The X-ray diffraction (XRD) patterns of the whole thin films series after thermal cycles are displayed in Fig. 1a. As can be seen, all patterns exhibited the main diffraction peaks corresponding to the CoSb_3_ skutterudite structure (*Im*3̄, *a* ≈ 9.04 Å) with high crystallinity as confirmed by cross-section Scanning Electronic Microscope (SEM) (Fig. 2a).^49^ Additional low-intensity peaks are observable in the sample T1 and can be attributed to residual free-Sb. The main thin films structures were confirmed by XRD refinement considering the CoSb_3_ skutterudite native structure. Low-reliability factor is obtained independently to the Te target power used during deposition, as presented in Table 1, and confirmed the systematic skutterudite phase formation with high purity. XRD pattern refinement allowed to determine the lattice parameter dependency with the Te target power and permitted to estimate the Te doping level in each film (Table 1 and Fig. 1b). Nonetheless, no conclusion about the atomic distribution of the Te in the Sb site and/or the void site can be drawn from the XRD refinement due to the limited resolution of a conventional XRD diffractometer. The increase in the cell parameter led us to the assumption that Sb substitution by Te occurred in accordance with the previous report on bulk but it is not excluded that the Te partially filled the void position in skutterudite.^38,40^ Further analysis, using transmission electronic microscope (TEM), is required to elucidate the real position of Te. The pseudo-linear and large increase of the lattice parameter *versus* Te target power is observable for T3 and T4 in contrast to T2. According to the paper of Li *et al.*, the cell parameter dependence with Te doping level reaches a step at *a* = 9.053 Å corresponding to a Te substitution of 3.13 at%.^38^ Nevertheless, in the report of Nagamoto *et al.*, a solubility limit of 6.2 at% is proposed with corresponding cell parameters of *a* ≈ 9.049 Å.^17^ In our present study, the cell parameters drastically increased up to *a* = 9.110 Å for T4 suggesting that a higher doping level can be reached by the sputtering method. The composition analysis by Scanning Electron Microscopy using Energy Dispersive X-ray Spectroscopy (SEM-EDS) also sustained this hypothesis with a richest Te composition (>6.2 at%) for the sample T3 and T4 (Tables 2 and S2†). It revealed a clear tendency of increasing Te content with the increasing of the Te target power simultaneously with a decreasing of the Sb content following the main substitution between the Sb and the Te. Also, as can be seen from Table 2, the thickness of the samples increased slightly in good agreement with the theoretical larger deposition rate, provided by the increasing power of Te target, during co-deposition. Although the EDS analysis showed a Te concentration of 12.22% and 22.14% for T3 and T4 films, respectively, these are likely to include contributions from Sb_2_Te_3_ nano particles, as will be shown below. Nevertheless, the large lattice parameters of these samples indicate that the Te concentrations in the CoSb_3_ phase of sample T3 and T4 are much higher than the solubility limit so far reported for bulk samples.^17^

**Table 1:** Cell parameters at room temperature and reliability factors obtain from Rietveld refinement of CoSb_3_ doped Te thin films (T1, T2, T3, and T4) X-ray patterns (*λ*_Cu_ = 1.5406 Å)

*Im*3̄	*a* (Å)	*V* (Å^3^)	*χ* ^2^	*R*_F_	*R*_Bragg_
T1	9.0446	739.894	1.12	7.88	8.83
T2	9.0453	740.063	1.22	5.63	8.27
T3	9.0844	749.690	3.22	7.52	21.9
T4	9.1104	756.146	2.05	9.33	18.1

**Table 2:** Atomic content and thickness of CoSb_3_ doped Te thin films (T1, T2, T3 and T4)

	Co content (at%)	Sb content (at%)	Te content (at%)	Thickness (nm)
T1	19.89	80.11	—	940
T2	20.29	76.47	3.24	951
T3	18.20	69.58	12.22	1002
T4	15.82	62.04	22.14	1123

The substantial Te content affected the surface morphology as shown in Fig. 2. The undoped (T1) and low Te-doped (T2) films revealed a slightly rough surface composed of irregular particles in the range of 100 nm to 1 μm. These particles are assimilated to the crystallized CoSb_3_-skutterudite as highlighted by the broad diffraction peak (Fig. 1b inset). Nonetheless, the T3 and T4 films revealed a flatter surface covered by nanoscale particles. We ascribed these nanoparticles to a Sb_2_Te_3_ phase (*R*3̄*mH*, *a* ≈ 4.26 Å and *c* ≈ 30.46 Å) which was seeded between the larger CoSb_3_ particles during the deposition process. This assumption is supported by the XRD pattern of the T4 which exhibited low intensity and broad peaks located at 2*θ* ≈ 28.20° and 42.40° (Fig. 1a (#)). It can be assimilated to the highest intensity indexation (015) and (110) of the Sb_2_Te_3_ crystal structure. Moreover, the Sb–Te rich composition revealed by SEM-EDS analysis (Tables 2 and S2†) also supports this assumption. A recent study has shown that the RF-magnetron sputtering method is suitable for obtaining nanosize Sb_2_Te_3_ in the range of 5.8–19.6 nm depending on the annealing.^50^ The seeding of the Sb_2_Te_3_ phase is made propitious by the systematic free-Sb, as observed in the un-doped film T1, and associated with the oversupply of Te provided by the large target power used for T3 and T4 deposition.

**Fig. 3:**
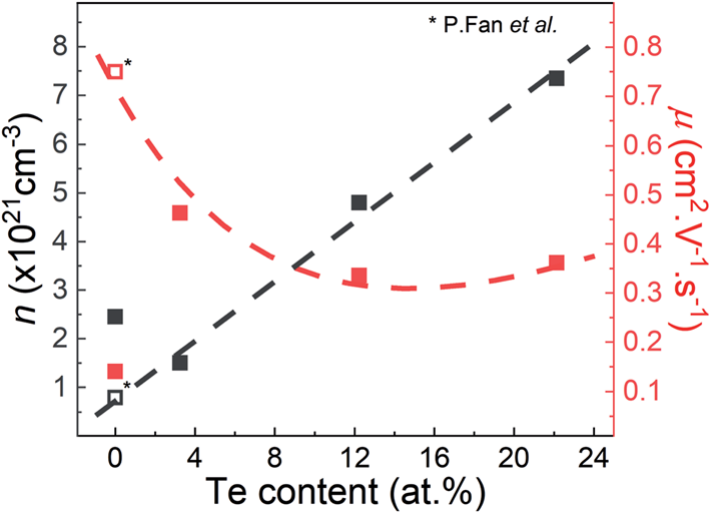
Carrier concentration and electron mobility at room temperature as a function of Te content of the CoSb_3_ doped Te thin films (T1, T2, T3, and T4) and reference data of CoSb_3_ thin film annealed at 518 K (asterisk).^31^

All thin films are characterized by n-type conduction with large carrier concentrations in the range of 10^21^ cm^−3^ as presented in Fig. 3. It indicates that the deposition process affected the film composition and promoted an intrinsic electron doping effect. According to the study of Zheng *et al.*, the presence of free-Sb in the undoped film T1 (Fig. 1a (*)) attested that an Sb deficiency involved in the CoSb_3_ film, which thereby induces additional electron carrier leading to the n-type conduction. As an example, the study of P. Fan *et al.* reported for CoSb_3_ thin film sample annealed at 518 K a carrier concentration of *n* ≈ 0.8 × 10^21^ cm^−3^ and an electronic mobility of *μ* ≈ 0.75 cm^2^ V^−1^ s^−1^ at RT (Fig. 3 (*)).^31^ But in the present study, it can be seen that the undoped film T1 is characterized by slightly larger carrier concentration (*n* = 2.45 × 10^21^ cm^−3^ at RT) and a significantly reduced electronic mobility (*μ* = 0.14 cm^2^ V^−1^ s^−1^). It suggests that our films are characterized by larger Sb deficiency. By using the data reported by P. Fan *et al.* as reference values, we can see that the carrier concentration significantly increases with the theoretical Te content up to *n* = 7.35 × 10^21^ cm^−3^ at RT for T4 according to the electron donor effect of the Te substitution.^38,39^ It is followed by a slight drop of the carrier mobility from *μ* = 0.463 cm^2^ V^−1^ s^−1^ to 0.362 cm^2^ V^−1^ s^−1^, respectively, for T2 and T4 (Fig. 3). The decrease of the electron mobility can likely to be related to the ionized impurity scattering effect enhancement related to the rise of the carrier concentration between the two samples. Other possibilities can be the alloy scattering and the potential fluctuation effect due to a non-uniform distribution of Te atoms over the lattice.

**Fig. 4:**
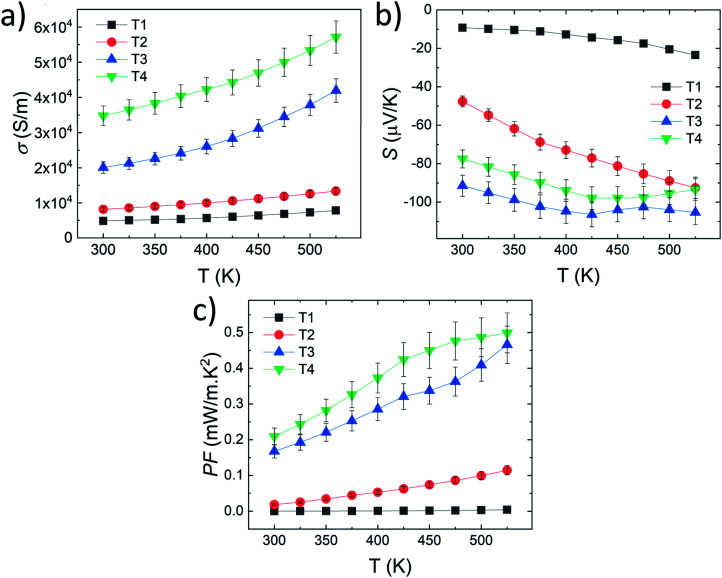
(a) Electrical conductivity *σ*, (b) Seebeck coefficient *S* and (c) power factor PF as the function of the temperature of the CoSb_3_ doped Te thin films (T1, T2, T3, and T4).

The electronic mobilities remained low, by comparison with the bulk CoSb_3_ skutterudite, and drastically affected the electrical transport properties as presented in Fig. 4a. The undoped film T1 exhibited semiconducting behavior with low electrical conductivity *σ* far from the native properties of the CoSb_3_ bulk sample.^13,14,18,21^ This electrical transport behavior is typical for a heavily doped semiconductor. Therefore, it is consistent with the large carrier concentration and the reduced electronic mobility due to the thin film formatting effect and small grain size in all films.^33,34^ The Te doping effect allowed to improve the electrical conductivity in the whole temperature range by the increase of the carrier concentration. As can be seen in Fig. 4a, electrical conductivity gradually increased with Te content from *σ* = 4.9 × 10^3^ S m^−1^ to 3.5 × 10^4^ S m^−1^ at 300 K, respectively, for T1 and T4. The presence of nanosize Sb_2_Te_3_, in T3 and T4 films, is assumed to have a negligible effect on the electrical properties considering the small amount detected and the poor electrical mobility of this phase.^50^

The Seebeck coefficient measurement confirmed that the whole series is characterized by n-type conduction with a negative Seebeck coefficient. The undoped film T1 is represented by low values in the temperature range (*S* = −9.2 to −23.5 μV K^−1^ from 300 to 525 K; Fig. 4b) comparable with several reports on undoped CoSb_3_ thin films.^31,33,34^ However, the Te doping induced a significant increase of the absolute value of the Seebeck coefficient up to *S* = −91.5 μV K^−1^ at 300 K for example in T3. The larger carrier concentration of T1 (*n* = 2.45 × 10^21^ cm^−3^ at RT) compared to T2 (*n* = 1.50 × 10^21^ cm^−3^ at RT) follow the predictive Pisarenko plot of the CoSb_3_-skutterudite presented in the report of Tang *et al.*^51^ Indeed, it is consistent with the multiple band transport model proposed for the heavily doped n-type CoSb_3_ skutterudite and explains the larger absolute Seebeck coefficient of the low Te-doped film T2 compared to the undoped film T1. However, it suggests an additional feature leading to the large thermopower in T3 and T4. As presented before, the large Te amount leads to a change of the crystal structure. The lattice parameter values are significantly increased for the films T3 and T4 (*a* ≥ 9.0844 Å at 300 K, Table 1) by comparison with T1 and T2 (*a* ≤ 9.0453 Å). The large Te amount can be considered to have induced a crystal field modification by the variation of the Sb–Co–Sb angle and bond length, as described in the report of Hanus *et al.*^52^ The ‘lattice effect’ can contribute to a band convergence in the CoSb_3_ electronic structure and be cumulated with carrier density effect for promoting a large Seebeck coefficient.

**Fig. 5:**
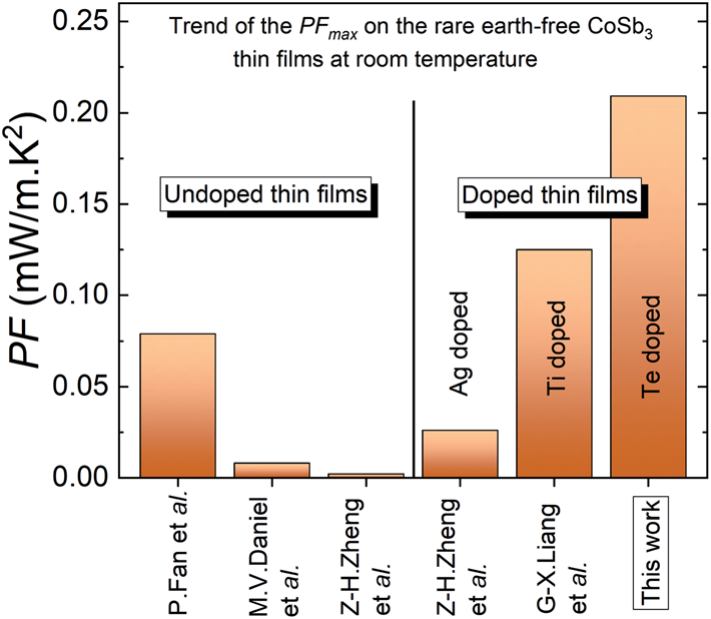
The trend of the highest PF obtained on the rare earth-free CoSb_3_-skutterudite thin films at room temperature.^24,26,30,32,34^

Correlated with the conductivity improvement, the Seebeck enhancement leads to a significant improvement of the PF of the thin film by 2 orders of magnitude in T4 up to a value of PF = 0.21 mW m^−1^ K^−2^ at 300 K (PF = 0.50 mW m^−1^ K^−2^ at 525 K) as presented in Fig. 4c. To the best of our knowledge, this is the highest power factor obtained in rare earth-free CoSb_3_-skutterudite thin films near room temperature and represent an improvement of at least 260% compared to the report of Fan *et al.* on flexible substrates and 840% considering the recent report on Ag-doped CoSb_3_ skutterudite thin film at room temperature.^24,31^ The drastic PF improvement, induced by the Te doping effect, constitutes a new record considering the last few years trend of the highest PF obtained on the rare earth-free CoSb_3_-skutterudite thin films at room temperature (Fig. 5).

To probe the thin films formatting effect in the thermal transport performance near room temperature, the thermal conductivity of the thin films was measured by the thermoreflectance method with front detection (S3),^47,48^ using a customized focused system.^46,53,54^ The results of the calculated total *κ* and lattice *κ*_latt_ thermal conductivity are summarized in Table 3. The electronic thermal conductivity *κ*_elec_ contribution seems negligible compared to the lattice thermal conductivity contribution. The pristine sample (T1) presented a significantly reduced *κ*_latt_ of 3.8 W m^−1^ K^−1^ @ 300 K compared to the corresponding values reported on undoped bulk samples (*κ*_bulk_ ≈ 8–9 W m^−1^ K^−1^ @ 300 K).^38,55–57^ According to the theoretical prediction of Shiga *et al.*, a significant *κ* reduction of CoSb_3_ thin film should occur for thin films thickness bellow 500 nm.^58^ However, the present thin films are in the region over 800 nm (Table 2) which confirmed that even ‘thick’ film formatting can efficiently reduce the thermal transport in the CoSb_3_ phase by the enhancement of boundary scattering in the surface. This effect can be cumulated with the small grain size (<500 nm) and a higher fraction of grain boundaries (Fig. 2b) which can contribute to lower the thermal conductivity as reported in nanostructured CoSb_3_.^59,60^ Te doping provided an additional reduction of the *κ*_latt_, as already reported in bulk materials. Considering that the samples have a high carrier concentration and low mobility, which indicates a high effective mass, we speculate that phonon-electron scattering can be induced by Te doping and also might have a non-negligible role.^21,38^ Ultimately we have obtained a minimum value of *κ*_latt_ = 2.2 W m^−1^ K^−1^ @ 300 K for T3. The slightly increased value of *κ*_latt_ = 2.9 W m^−1^ K^−1^ @ 300 K for the T4 film with heavy Te doping might be due to the Sb_2_Te_3_ phase which affected the front detection of the thermoreflectance analysis by increasing the interfacial thermal resistance. The final figure of merit varied between *zT* = 0.02–0.04 near room temperature (300–375 K) and increased up to *zT* = 0.08–0.1 at 525 K for the T3 and T4 thin films.

**Table 3:** Thermal diffusivity, calculated density, and specific heat, and the total/lattice thermal conductivity of CoSb_3_ doped Te thin films (T1, T2, T3, and T4)

At 300 K	Thermal diffusivity (m^2^ s^−1^)	Density (kg m^−2^)	Specific heat (J (kg^−1^ K^−1^))	Thermal conductivity (W (m^−1^ K^−1^))	Lattice thermal conductivity (W (m^−1^ K^−1^))
T1	2.59 × 10^−6^	7616	235.0	3.8	3.78
T2	1.53 × 10^−6^	7614	235.0	3.2	3.15
T3	1.50 × 10^−6^	7516	235.0	2.4	2.23
T4	2.14 × 10^−6^	7452	235.0	3.2	2.94

## Conclusion

In this study, Te-doped CoSb_3_-skutterudite thin films were prepared for the first time by using the co-sputtering of a CoSb_3_ target and pure Te target. Tellurium doping was confirmed through XRD refinement and SEM-EDS analysis. We revealed that the sputtering method allowed to overcome the Te solubility limit reported of 6.2 at% on bulk samples. The electron doping provided with the substitution of Sb by Te leads to an increase in the carrier concentration and enhanced the poor electrical conductivity of the CoSb_3_ thin films up to *σ* ≈ 3.50 × 10^4^ S m^−1^ at 300 K for the richest Te content. Simultaneously, the Seebeck coefficient is increased up to *S* = −93.4 μV K^−1^ at 525 K for this heavily Te-doped thin films which provided the best power factor currently reported in rare earth-free CoSb_3_ skutterudite films of PF = 0.21 to 0.50 mW m^−1^ K^−2^, respectively, at 300 K and 525 K. Furthermore, we determined the thermal conductivity of the CoSb_3_-skutterudite thin films (*κ* = 3.8 W m^−1^ K^−1^ @ 300 K for the undoped film) which highlighted that even thick film formatting can efficiently reduce the large thermal conductivity of the phase. Finally, we showed that the co-deposition method is suitable for preparing highly doped-CoSb_3_ thin films.

## Conflicts of interest

The authors declare no conflict of interest.

## Supplementary Material

RA-010-D0RA02699A-s001
