# Supplementary material for: Drastic power factor improvement by Te doping of rare earth-free CoSb3-skutterudite thin films
Source: RSC Adv. 2020 Jun 3;10(36):21129–35. doi: 10.1039/d0ra02699a (PMC9054351; doi:10.1039/d0ra02699a)
Supplement: RA-010-D0RA02699A-s001 [file RA-010-D0RA02699A-s001.pdf]

Electronic Supplementary Material (ESI) for RSC Advances.
This journal is © The Royal Society of Chemistry 2020

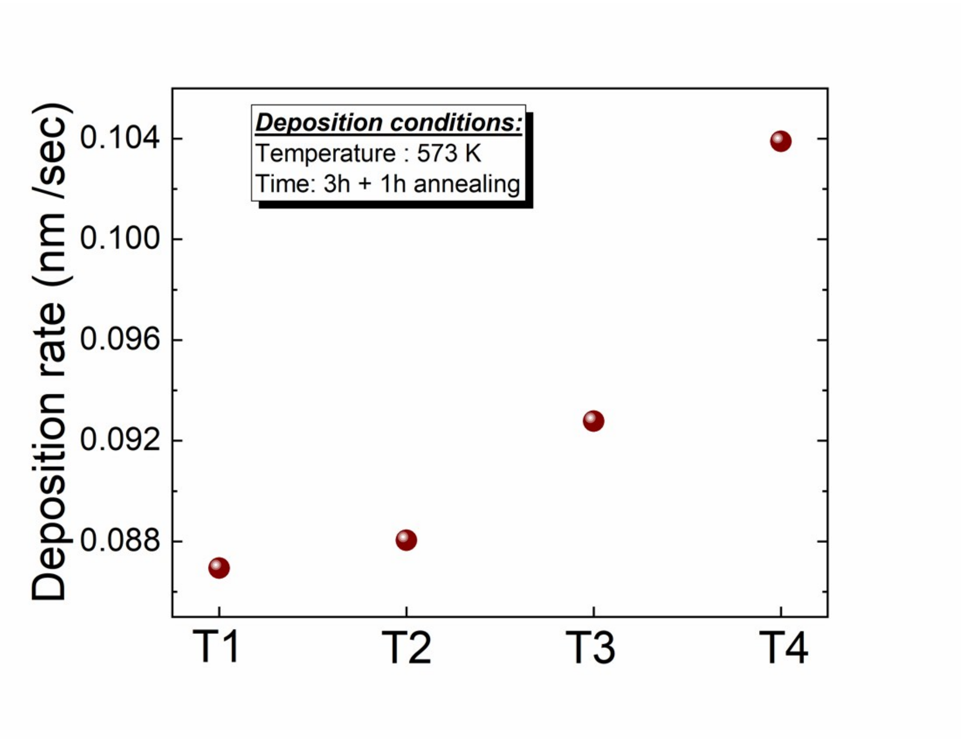

S1. Deposition rates of the $CoSb_3$ doped Te thin films (T1, T2, T3, and T4)

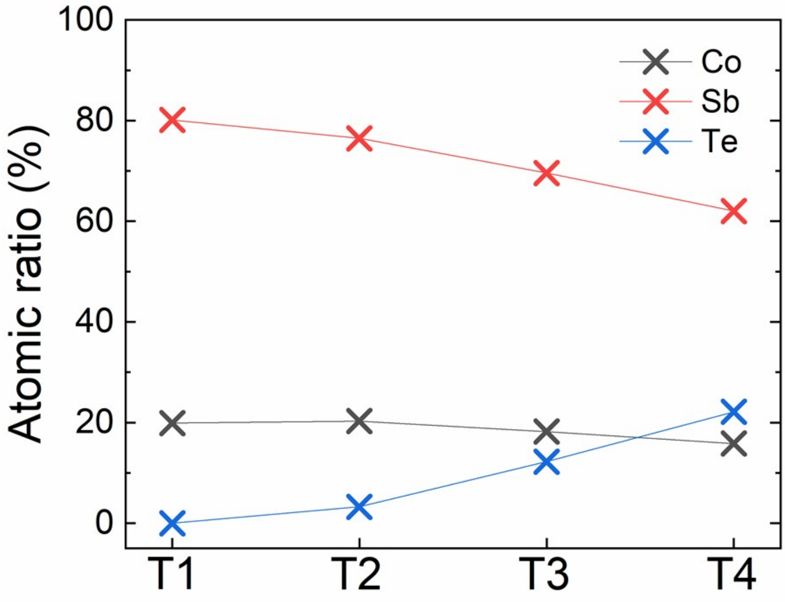

S2. Atomic ratio measurements by SEM-EDS of the $CoSb_3$ doped Te thin films (T1, T2, T3, and T4)

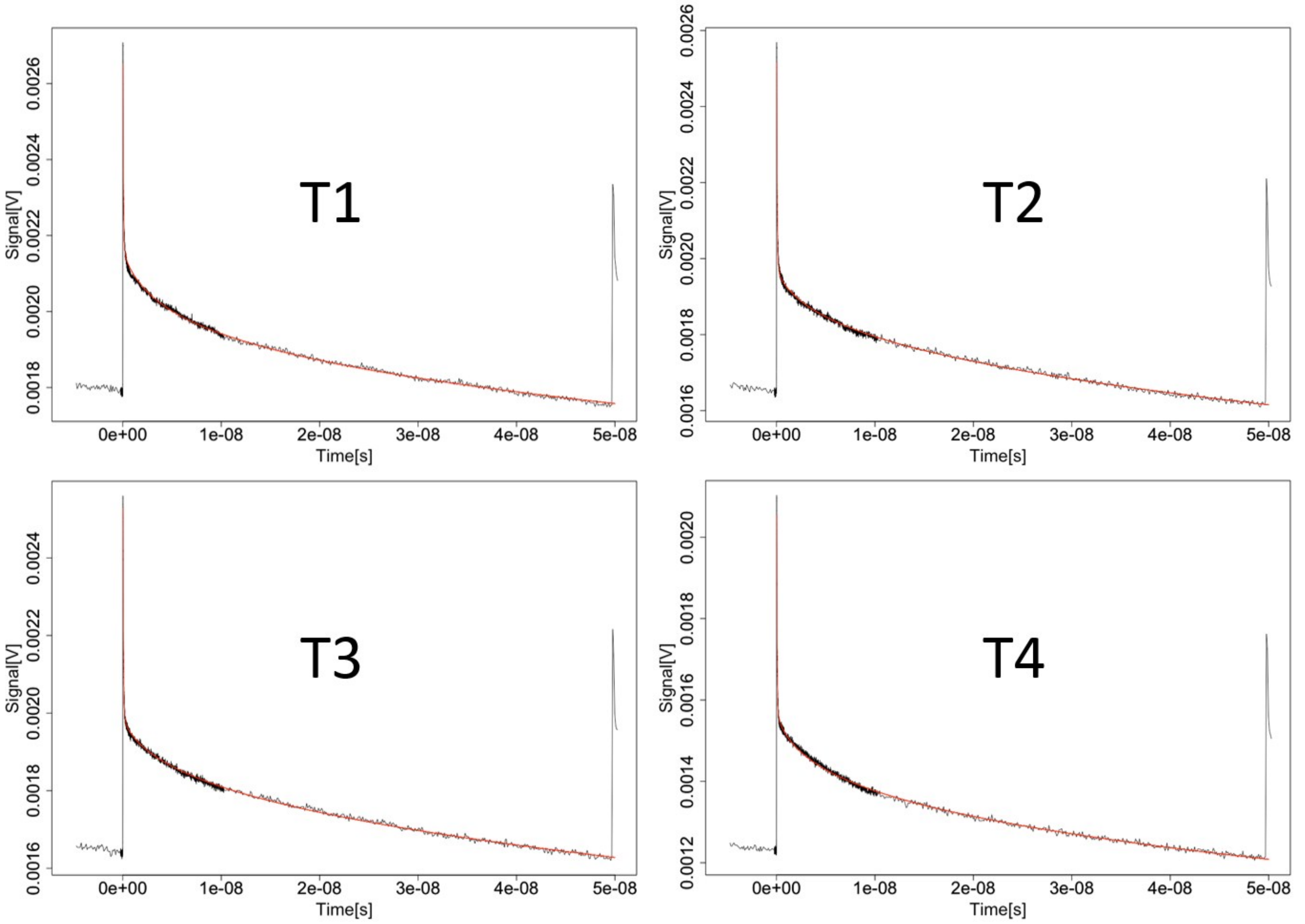

S3. Thermoreflectance measurements in front detection and resulting signal fitting of the $CoSb_3$ doped Te thin films (T1, T2, T3, and T4)
